# Toll-like receptor 4 signaling in intracerebral hemorrhage-induced inflammation and injury

**DOI:** 10.1186/1742-2094-10-27

**Published:** 2013-02-17

**Authors:** Huang Fang, Peng-Fei Wang, Yu Zhou, Yan-Chun Wang, Qing-Wu Yang

**Affiliations:** 1 Department of Neurology, Second Affiliated Hospital and Xinqiao Hospital, Third Military Medical University, Xinqiao Zhengjie No.183, Shapingba District, Chongqing 400037, China

**Keywords:** Toll-like receptor 4, Intracerebral hemorrhage, Inflammation, Hematoma resolution

## Abstract

Intracerebral hemorrhage (ICH) is a common type of fatal stroke, accounting for about 15% to 20% of all strokes. Hemorrhagic strokes are associated with high mortality and morbidity, and increasing evidence shows that innate immune responses and inflammatory injury play a critical role in ICH-induced neurological deficits. However, the signaling pathways involved in ICH-induced inflammatory responses remain elusive. Toll-like receptor 4 (TLR4) belongs to a large family of pattern recognition receptors that play a key role in innate immunity and inflammatory responses. In this review, we summarize recent findings concerning the involvement of TLR4 signaling in ICH-induced inflammation and brain injury. We discuss the key mechanisms associated with TLR4 signaling in ICH and explore the potential for therapeutic intervention by targeting TLR4 signaling.

## Introduction

Intracerebral hemorrhage (ICH) is the least treatable type of stroke and has devastating consequences [1]. Hemorrhagic strokes account for 15% to 20% of all strokes and are associated with high mortality and morbidity [2,3]. Primary damage caused by ICH occurs within the first few hours after the onset of bleeding and is mainly due to formation of hematomas, which compress adjacent tissues, thus destroying them [4]. Many patients with ICH deteriorate progressively with no sign of hematoma expansion, suggesting that secondary damage following ICH plays a critical role in neurological deterioration [5,6]. Several lines of evidence show that secondary damage involves inflammation, cerebral edema, and cellular apoptosis, ultimately leading to blood–brain barrier disruption and massive brain cell death [4,7-10]. The molecular mechanisms of secondary damage after ICH have not been well-established, but they may represent novel therapeutic targets to prevent further brain injury.

Secondary damage following ICH is triggered by the presence of intraparenchymal blood, which subsequently activates cytotoxic, oxidative and inflammatory pathways [4,10]. The toxic effects of extravasated blood result mainly from blood components, including red blood cells (RBCs), coagulation factors, complement components and immunoglobulins [4,5,10]. Thrombin, a serine protease produced rapidly after ICH onset, contributes to edema formation and blood–brain barrier damage in early brain injury, and activates the cytotoxic, excitotoxic and inflammatory pathways that are involved in secondary injury following ICH [5,10,11]. Hemoglobin (Hb) released from RBC lysis is a potent cytotoxic chemical that generates free radicals and oxidative damage, causing death of surrounding cells [5,10,12,13]. Hemin, the oxidative form of heme, plays a critical role in Hb-induced brain injury following ICH [5]. Hemin exerts its neurotoxic effects via release of excessive iron, depletion of glutathione and production of free radicals [14]. In addition, an inflammatory response occurs after ICH, which aggravates ICH-induced brain injury, leading to further tissue damage, blood–brain barrier disruption and edema [4,15,16]. The inflammatory mechanisms involved in progression of ICH-induced brain injury include activation of microglial cells, infiltration of inflammatory cells and production of cytokines and chemokines. Neutrophils are believed to contribute to brain injury after ICH [17,18]. Depletion of neutrophils reduced blood–brain barrier disruption, axon injury and inflammation in a rat model of ICH [18] and was found to prevent tissue plasminogen activator (tPA)-induced ICH in a rat model of cerebral ischemia [17]. Neutrophils may damage brain tissues by producing reactive oxygen species (ROS) and releasing proinflammatory cytokines and matrix metalloproteinases (MMPs) [19,20]. Several lines of evidence show that activation of innate immunity and inflammatory responses contributes to the pathogenesis of secondary injury after ICH [4,5,10,16].

Toll-like receptors (TLRs) belong to a large family of pattern recognition receptors that play a key role in innate immunity and inflammatory responses [21,22]. Thirteen mammalian TLRs have been identified in mice, eleven of which are also found in humans. They recognize distinct pathogen-associated molecular patterns from diverse organisms, including viruses, bacteria, mycobacteria, fungi and parasites [23,24]. In addition, TLRs also recognize damage-associated molecular patterns and mediate host inflammatory responses to injury [22]. TLR1, TLR2, TLR4, TLR5, TLR6 and TLR10 are distributed on the cell surface, and other TLRs (TLR3, TLR7, TLR8, and TLR9) are expressed in the intracellular endosomes [22,25]. TLR consists of leucine-rich repeats in the extracellular ectodomains that bind various ligands, as well as intracellular Toll-interleukin 1 receptor (TIR) domains that recruit intracellular adaptor proteins, including myeloid differentiation factor 88 (MyD88), TIR domain-containing adaptor-inducing interferons (TRIFs), TIR domain-containing adaptor protein (TIRAP) or TRIF-related adaptor molecule (TRAM). TLR signaling pathways include at least a MyD88-dependent pathway common to all TLRs except TLR3, as well as a MyD88-independent pathway selective to TLR3 and TLR4 [22,26,27]. In the MyD88-dependent pathway, MyD88 activates signal transduction molecules, including interleukin (IL)-1R-associated kinases (IRAKs), tumor necrosis factor (TNF) receptor-associated factor 6 (TRAF6) and transforming growth factor (TGF)-β-activated kinase (TAK1), ultimately leading to activation of nuclear factorκB (NF-κB) and expression of proinflammatory cytokines. In the MyD88-independent pathway, TRIF activates signal transduction molecules including TANK-binding kinase 1 (TBK1) and interferon regulatory factor 3 (IRF3), ultimately leading to expression of interferonβ (IFN-β) [26]. Our recent study shows that both the MyD88 and TRIF signaling pathways are involved in TLR4-mediated inflammatory responses after ICH [28].

It has been reported that TLR4 is upregulated in a rat model of ICH [29,30] and that its signaling pathway contributes to poor outcome after ICH [31]. TLR4 is activated by many endogenous ligands, such as heme and fibrinogen [32,33], which are produced in the brain after ICH. Our recent *in vivo* study shows that activation of TLR4 by heme causes ICH-induced inflammatory injury via the MyD88/TRIF signaling pathway and that effective blockade of TLR4 by its antibody suppresses ICH-induced inflammation [28]. Thus, the TLR4 signaling pathway could be a promising therapeutic target for ICH treatment.

TLR4 is expressed in microglia, the resident macrophages of the brain. Microglia are activated within minutes after ICH [34,35] and subsequently release chemotactic factors to recruit hematogenous phagocytes to the hemorrhagic areas. Timely clearance of the extravasated RBCs by activated microglia/macrophages can provide protection from local damage resulting from RBC lysis. Successful removal of injured cells can reduce secondary damage by preventing discharge of injurious proinflammatory cell contents. Resolution of hematoma and inhibition of inflammation are considered potential targets for ICH treatment [5,10,36,37]. In this review, we highlight the roles of TLR signaling pathways in ICH and discuss their potential as therapeutic targets.

### Innate immunity and inflammation in the pathogenesis of ICH

Microglial cells are activated within minutes after the onset of ICH [34,35]. Activated microglial cells undergo morphological and functional changes that include enlargement and thickening of processes, upregulation of proinflammatory proteins, and behavioral changes, including proliferation, migration and phagocytosis [10,20]. The primary neuroprotective role of activated microglia is to clear the hematoma and damaged cell debris through phagocytosis, providing a nurturing environment for tissue recovery. However, accumulating evidence has shown that microglial activation contributes to ICH-induced secondary brain injury by releasing a variety of cytokines, chemokines, free radicals, nitric oxide and other potentially toxic chemicals [16,34,38,39]. In addition, several studies have shown that inhibition of microglial activation reduces brain damages in animal models of ICH [39-41]. Microglial inhibitors, such as minocycline and microglia/macrophage inhibitory factors (tuftsin fragment 1–3), reduce ICH-induced brain injury and improve neurological function in rodents [40-45]. Clearly, microglial activation mediates ICH-mediated brain injury.

Besides microglia, other blood-derived inflammatory cells, such as leukocytes and macrophages, are also activated after ICH and contribute to ICH-induced brain injury [16]. Neutrophil infiltration occurs less than 1 day after the onset of ICH, and the infiltrating neutrophils die by apoptosis within 2 days [35,46]. Dying leukocytes can cause further brain injury by stimulating microglia/macrophages to release proinflammatory factors [16]. Activated macrophages are indistinguishable from resident microglia in morphology and function [20]. Similar to activated microglia, activated leukocytes and macrophages release a variety of cytokines, chemokines, free radicals and other potentially toxic chemicals [16,20,34].

Cytokines are well-known to be associated with inflammation and immune activation [47]. Although cytokines are released by many cells, including microglia/macrophages, astrocytes and neurons, the major sources of cytokines are activated microglia/macrophages [48]. Many studies have shown that two major proinflammatory cytokines, TNF-α and interleukin1β (IL-1β), exacerbate ICH-induced brain injury. After ICH, TNF-α is significantly increased both *in vivo* and *in vitro*[16,28,34,49], which may contribute to brain edema formation and brain injury in animal models of ICH [49,50]. Consistent with animal studies, clinical studies support the proposition that TNF-α contributes to ICH-induced brain injury. Plasma TNF-α has been shown to correlate with the magnitude of the perihematomal brain edema in patients with ICH [51]. Single-nucleotide polymorphisms in the TNF-α gene promoter are associated with spontaneous deep ICH [52]. Similarly, IL-1β has been found to be upregulated after ICH in an animal model and to produce detrimental effects, including brain edema and blood–brain barrier disruption [28,53,54].

NF-κB, a transcription factor involved in inflammatory responses, is also activated after ICH, leading to upregulation of gene expression that contributes to brain injury [34,55]. Activation of NF-κB occurs within minutes and lasts for at least 1 week after the onset of ICH [56]. NF-κB is a key regulator of many proinflammatory cytokines, such as TNF-α and IL-1β, in various pathological conditions, including ICH [16,28,55,57]. The activity of NF-κB correlates with perilesional cell death after ICH in rats [58]. Activation of NF-κB is positively associated with the progression of apoptotic cell death in patients with ICH [59]. Therefore, understanding the signaling mechanisms underlying ICH-induced NF-κB activation may facilitate identification of therapeutic targets.

Several lines of evidence have shown that NF-κB is activated by RBCs and plasma via signaling pathways involving free radicals, cytokines and glutamate receptors [55]. It is well-known that TLR signaling pathways can lead to NF-κB activation, resulting in production of proinflammatory cytokines [27,60,61]. Increasing evidence has shown that TLR signaling pathways play an essential role in sterile inflammatory diseases in the central nervous system [62-64]. Herein we review recent advances in TLR4 signaling pathways in ICH-induced inflammatory brain injury.

### TLR4 signaling in ICH-induced inflammatory brain injury

TLRs, especially TLR4, are involved in the inflammatory responses and neuronal damage associated with cerebral ischemia [22,65]. Expression of TLR4 is upregulated in a mouse model of transient cerebral ischemia [66]. In ischemic brain injury, TLR4-deficient mice show significant suppression of inflammatory cytokine expression, including IRF1, inducible nitric oxide synthase, cyclooxygenase2, MMP9, and IFN-β [67]. TLR4-knockout mice have significantly smaller infarct volumes and better neurological function than wild-type (WT) mice [66,67]. Similar to cerebral ischemia, TLR4 expression is significantly increased after ICH, and TLR4-knockout mice demonstrate improved neurological function after ICH [28-31]. These animal studies agree with recent clinical findings that increased expression of TLR4 is associated with poorer functional outcome and greater residual volume in patients with ICH [68]. However, although TLR4 participates in both cerebral ischemia- and ICH-induced brain injuries, the signaling pathways involved are different. Both the MyD88 and TRIF pathways are involved in TLR4-mediated brain injury in ICH [28], whereas only MyD88 is involved in ischemia [69]. Therefore, the roles of TLR4 in ICH and cerebral ischemia may be different.

The roles of TLR4 in inflammation and neurological impairment following ICH have been studied recently, using TLR4-knockout mice, by our research group and others [28,31]. Compared to WT mice, after ICH, TLR4-knockout mice exhibited significantly decreased brain water content [28] and neurological deficits [28,31]. Furthermore, the perihematomal region in TLR4-knockout mice had lower levels of infiltrating inflammatory cells, including macrophages [28], leukocytes and monocytes [31], as well as lower levels of inflammatory cytokines, such as TNF-α, IL-1β and IL-6, and decreased NF-κB activity [28]. In addition, Sansing *et al*. [31] reported increased gene expression of CD36, CSF2 and CX3CL1 in TLR4-knockout mice compared to WT mice. Taken together, these studies demonstrate that TLR4 activation is involved in ICH-induced neurological deficits and contributes to the detrimental inflammatory response.

TLR4 is expressed in various cell types in the central nervous system, including microglia, neurons and astrocytes, as well as in peripheral blood cells, such as leukocytes, macrophages and platelets [22,28,30,33,70,71]. After ICH, TLR4 mRNA and protein expression is significantly increased by approximately 2 to 6 hours, peaks at day 3, declines somewhat at day 5, but remains elevated relative to baseline even at day 7 [28-30]. Though ICH induces upregulation of TLR4 expression in neurons, astrocytes and microglia, TLR4 is predominantly expressed in CD11b-positive microglial cells in mice [28]. In addition, TLR4 contributes to reduced recruitment of neutrophils and monocytes in the perihematomal area after ICH in TLR4-deficient mice [31]. Therefore, resident microglia in the brain as well as peripheral infiltrating leukocytes and monocytes are likely involved in the roles of TLR4 in ICH-induced brain injury.

Microglial activation in response to ICH contributes to ICH-induced brain injury by releasing cytokines, and inhibition of microglial activation has been shown to improve neurological function in animal models of ICH [38-41]. Recently, we investigated the role of TLR4 in microglial activation following ICH [28]. Exogenous hemin treatment of cultured microglia increases expression of TLR4, as well as proinflammatory cytokines, such as TNF-α, IL-1β and IL-6. This effect is completely abolished by knockout of TLR4 or treatment with anti-TLR4 antibodies, suggesting that TLR4 mediates hemin-stimulated microglial activation [28]. In addition, hemin-induced expression of proinflammatory cytokines TNF-α, IL-1β and IL-6 is completely blocked in TLR4-knockout mice [28]. These data demonstrate that TLR4 signaling mediates heme-induced inflammatory injury, possibly by activating microglial cells and subsequently releasing proinflammatory cytokines. However, Sansing *et al*. [31] reported that upregulation of IL-1β and IL-6 genes in the perihematomal region did not significantly differ between TLR4-knockout and WT mice, suggesting that TLR4 signaling may not be involved in the transcriptional regulation of proinflammatory cytokines in perihematomal inflammation. Alternately, it may not be possible to detect upregulation of proinflammatory cytokine genes in certain cell types, such as microglia, when the entire perihematomal region is used [31].

Using blood transfer experiments in which TLR4-deficient blood was injected into the brains of WT mice and WT blood into brains of TLR4-deficient mice, Sansing *et al*. [31] found that TLR4 signaling within the hemorrhage mediated the inflammatory response and contributed to ICH-induced neurological injury. Activation of TLR4 on leukocytes or platelets within the hemorrhage, but not on resident cells, promoted inflammation and resulted in poor functional outcomes. This study clearly demonstrated that TLR4 signaling on peripheral blood cells plays a critical role in ICH-induced brain injury.

Therefore, TLR4 signaling on resident microglia and on blood cells within the hemorrhage is critical for ICH-induced inflammatory injury. Because TLR4 is expressed on resident microglia which are activated within minutes after ICH [34,35], the TLR4 signaling on microglia probably initiates ICH-induced inflammatory injury, causing release of inflammatory cytokines and infiltration of neutrophils. Significant upregulation of TLR4 expression occurs at approximately 2 to 6 hours after ICH [28-30], accompanied by the appearance of infiltrating neutrophils in the hematoma (at approximately 4 hours after ICH) [46]. TLR4 signaling in the neutrophils may further mediate release of proinflammatory cytokines, which contributes to the detrimental inflammatory response [17,18]. There are abundant TLR4 activators in the perihematomal region, including heme [32], fibrinogen [33] and myeloid-related proteins 8 and 14, which are released from degranulating neutrophils [72]. These endogenous activators can stimulate TLR4 on leukocytes, leading to inflammatory injury.

### TLR4 signaling pathways in ICH

TLRs are a group of Class I transmembrane proteins that consist of ectodomains, transmembrane domains, and intracellular TIR domains. TLRs recognize and bind various ligands via leucin-rich repeats in the extracellular ectodomains. The receptor-ligand binding results in conformational changes of the receptor, subsequently leading to recruitment of intracellular adaptor proteins, including MyD88, TRIF, TIRAP, or TRAM [22]. Once recruited, these adaptors initiate downstream signaling events, which ultimately lead to activation of transcription factors, such as NF-κB, and subsequent expression of various proinflammatory cytokines, such as IL-6, TNF-α and IL-1 [22,27].

TLR4 interacts with two distinct adaptor proteins (MyD88 and TRIF) and therefore activates two parallel signaling pathways to initiate the activation of transcription factors that regulate expression of proinflammatory cytokine genes [22,26,73]. The MyD88-dependent pathway, common to all TLRs, is essential for activation of NF-κB and production of inflammatory cytokines [26,74]. TRIF is essential for the TLR3- and TLR4-mediated MyD88-independent pathway, which involves the activation of IRF-3, leading to expression of IFN-β [26,27]. Recently, we found that both MyD88 and TRIF signaling pathways are involved in TLR4-mediated inflammatory responses after ICH [28]. Deletion of MyD88 or TRIF in transgenic mice leads to improved neurological function and reduced cytokine release and macrophage infiltration following ICH. After ICH in TLR4-knockout mice, MyD88 and TRIF expression are reduced, further demonstrating the role of these factors in TLR4-mediated ICH sequelae. Furthermore, the ICH-induced increase in NF-κB activity is significantly lower in TLR4-knockout mice than that in WT mice, suggesting that TLR4 mediates ICH-induced inflammation via activation of NF-κB to regulate expression of inflammatory cytokines.

The MyD88 pathway of TLR4 signaling activates not only the NF-κB pathway but also the activating protein1 (AP-1) pathway. AP-1 is a dimeric protein composed of members of the Jun, Fos, and α-fetoprotein families of proteins [75]. AP-1 activation leads to the expression of many proinflammatory mediators, such as MMPs, proteases, and cytokines such as IL-1 and IFN [76,77]. It is well-documented that these proinflammatory mediators are increased after ICH [16,20]. For example, MMPs, including MMP2, MMP3, MMP9, and MMP12, have been reported to be upregulated after ICH as a result of their activation by proteases such as plasmin and tPA [20,78,79]. However, the role of AP-1 in ICH has not been explored yet. AP-1 activation in TLR4 signaling is mainly mediated by mitogen-activated protein kinases (MAPKs), including c-Jun N-terminal kinase (JNK), p38 and ERK [76]. JNK, a stress-activated kinase that mediates apoptosis in neurons and microglia, is activated after ICH, and inhibition of the JNK pathway results in a significant decrease in edema and hematoma volume in mice after ICH [80]. Therefore, proapoptotic signals may play a role in ICH-induced inflammatory injury. However, it remains unknown whether ILR4 can mediate ICH-induced inflammatory injury via AP-1 activation.

Interestingly, lively and Schlichter showed that the expression of inflammatory mediators after ICH is age-dependent [81]. They examined 27 genes, including TLR4, and found that 18 of the 27 genes were different in expression levels or timing between young adult and aged rats [81]. The delayed expression of TLR4 was observed in aged rats, accompanied by a delayed and/or decreased expression of many proinflammatory cytokines such as IL-1β and IL-6 [81]. The TLR4 signaling pathways involved in age-related expression of proinflammatory cytokines is not well-understood. It has been reported that lipopolysaccharide (LPS)-induced production of TLR4-mediated proinflammatory cytokines is age-dependently decreased, accompanied by a decrease in the expression of MAPKs, but not the surface expression of TLR4 [82]. Decreased TLR4/MPAK signaling pathway may be responsible for age-related decrease in the production of inflammatory cytokines.

Because the brain is a sterile organ without any pathogens derived from bacteria or viruses, the endogenous molecules released after ICH become TLR stimulators. Several endogenous molecules have been reported as TLR4 ligands, including heme, fibrinogen, high-mobility group protein B1 (HMGB1), heat shock proteins, hyaluronan, oxidized low-density lipoprotein and amyloid β [32,33,69,83-87]. However, the molecular mechanisms underlying activation of TLR4 signaling pathways by these different ligands are not completely understood. Clearly, different TLR4 ligands can activate distinct signaling pathways. For example*, in vivo* animal studies show that heme triggers a TLR4 signaling pathway involving both MyD88 and TRIF [28], whereas HMBG1 initiates only the MyD88 pathway [69], and monophosphoryl lipid A, a low-toxicity derivative of LPS, activates only the TRIF pathway [88]. It remains unclear how different TLR4 ligands selectively activate distinct signaling pathways. However, different TLR4 receptor conformations induced by binding of different TLR4 ligands may contribute to the pathway-specific activation [89]. The ligand-biased signaling is well-known for G protein-coupled receptors, such as β-adrenergic receptors [25,90]. Understanding the mechanisms of biased signaling can provide leads for designing more specific drugs.

Many endogenous TLR4 ligands are known to be released during ICH. Some TLR4 ligands are crucial for activating TLR4 to trigger ICH-induced inflammation and inflammatory cytokine expression [28,31,91,92]. Heme, released from RBC lysis after ICH, is essential for TLR4-mediated inflammation because it potentiates microglial activation and increases cytokine expression [28]. Fibrinogen within the clots after ICH is also critical for activation of TLR4 on platelets or leukocytes within the hemorrhage, which contributes to poor outcome after ICH [31]. HMGB1, known to be essential for ischemic brain injury [65], is reported to be upregulated in the microglia after subarachnoid hemorrhage [91], and the HMGB1 inhibitor glycyrrhizin attenuates ICH-induced brain injury [92]. Additional studies of the roles of endogenous TLR4 ligands in ICH are warranted.

A deeper understanding of TLR4 signaling pathways should enable development of potential therapeutic targets for prevention and treatment of ICH. There are two ways to block TLR signaling: direct blockade by removal of TLR ligands (e.g., heme) and inhibition of TLRs and their downstream signaling pathways. Effective removal of deposited blood and disintegrated cells by promoting hematoma clearance has been demonstrated to reduce ICH-induced neurological deficits in a mouse model of ICH [36,37]. Hematoma resolution could promote clearance of hemin, thus reducing hemin-mediated activation of TLR4 and subsequent inflammatory responses. In addition, effective blockade of TLR4 receptor using an antibody disrupted TLR4 signaling and has neuroprotective effects in a mouse model of ICH [28]. Therefore, effective blockade of TLR4 signaling pathway could be a potential therapeutic strategy for prevention and treatment of ICH.

### Blockade of TLR4 signaling as a potential target in the treatment of ICH

As TLR4 signaling plays an important role in ICH-induced inflammatory injury, TLR4 inhibition should be beneficial. TLR antagonists have been developed for a number of inflammatory and autoimmune diseases [93-95] and include anti-TLR antibodies, small-molecule antagonists screened from compound libraries, and antagonists derived from medicinal plants. However, the efficacy of TLR antagonists in ICH has not been well-studied to date.

We have found that a specific antibody (Mts50) blocked TLR4 signaling in a mouse model of ICH [28]. TLR4 antibody treatment significantly reduced cerebral water content and improved neurological function after ICH, similar to effects observed in TLR4-knockout mice, suggesting that blockade of the TLR4 receptor is a potential therapeutic approach in the treatment of ICH. The neuroprotective effects of the TLR4 antibody in ICH may be associated with inhibition of cytokine expression and macrophage infiltration. However, the effectiveness of the antibody on hemin-induced TLR4 activation in macrophages is controversial. We found that the TLR4 antibody effectively suppressed hemin-induced microglial activation in mice [28]. However, Figueiredo *et al*. reported that this antibody blocked only LPS-induced, but not hemin-induced, TLR4 activation in macrophages, suggesting that different TLR4 receptor conformations exist in response to different ligands [32]. The discrepancy between the two studies may be due to use of different reagents and animal models, and further studies are needed to evaluate the efficacy of the TLR4 antibody for ICH.

Many TLR4 antagonists have been reported to produce anti-inflammatory effects [93-95]. Some antagonists, such as curumin, 6-shogaol, isoliquiritigenin, and OSL07 (4-oxo-4-(2-oxo-oxazolidin-3-yl)-but-2-enoic acid ethyl ester), block TLR4 signaling by inhibiting homodimerization of TLR4 [96-99]. Other agents, such as sparstolonin B, auranofin, TAK-242, and M62812, have also been reported to block TLR4 signaling pathways selectively [100-103]. However, most of these agents were tested in an animal model of LPS-induced sepsis. The effects of these agents on ICH-induced inflammation have not yet been explored.

In addition to these antagonists, many agents with multiple pharmacological mechanisms have been found to have neuroprotective effects in animal models of ICH via inhibition of TLR4 signaling. For example, oxymatrine, which has anti-inflammatory, antioxidative, and antiapoptotic activities, suppresses TLR4 and NF-κB gene expression and decreases production of proinflammatory cytokines, such as IL-6, TNF-α, and IL-1β [104]. Ginkgolide B, a specific platelet-activating factor receptor antagonist, reduces neuronal cell apoptosis after traumatic brain injury in rats, possibly via inhibition of TLR4 signaling pathways [105]. Progesterone treatment inhibits TLR4 signaling pathways and reduces brain edema and blood–brain barrier impairment after subarachnoid hemorrhage in rats [106]. These agents are not specific TLR4 receptor blockers, but they can inhibit TLR4 signaling to reduce ICH-induced inflammatory injury.

Paradoxically, TLR4 activation with low doses of LPS prior to ischemic brain injury protects against subsequent severe ischemic injury [107-109]. The mechanisms of preconditioning by TLR4 activation in ischemic brain injury are not fully understood. Recent studies have shown that LPS preconditioning redirects TLR4 singling through the TRIF-IRF3 pathway, but not through the MyD88 pathway [109,110]. Enhanced IRF3 activity and increased anti-inflammatory IFN gene expression contribute to the beneficial effects of LPS preconditioning [109]. Though the suppression of NF-κB activity is suppressed in LPS preconditioning mice following ischemic injury, proinflammatory cytokine production does not change, suggesting that besides the TLR4 signaling pathway, other signaling cascades and transcription factors are involved in proinflammatory cytokine production during ischemic injury [109]. However, there have been relatively few studies examining the effects of preconditioning by TLR4 activation on ICH. It remains to be determined whether the TRIF/IRF3 pathway and enhanced anti-inflammatory IFN production are preferentially involved in preconditioning by TLR4 activation in ICH. It has been reported that progesterone inhibits TLR4/NF-κB signaling pathway and decrease proinflammatory cytokine production in rats following subarachnoid hemorrhage [106], suggesting that suppressed proinflammatory signaling may contribute to preconditioning by TLR4 activation in ICH. Therefore, suppressed proinflammatory signaling and/or enhanced anti-inflammatory IFN signaling may be associated with preconditioning by TLR4 activation in ICH. Additional studies of the mechanisms of preconditioning by TLR4 activation in ICH are warranted.

### Heme removal and hematoma resolution as potential targets in the treatment of ICH

RBC lysis occurs at approximately 24 hours after the onset of ICH and continues for the next several days, leading to release of cytotoxic hemoglobin [111]. Hemoglobin then degrades to hemin, the oxidative form of heme [14]. Hemin is gradually cleared by hematogenous phagocytes and resident microglia [10]. Once inside these cells, hemin is degraded by heme oxygenase (HO) into biliverdin and carbon monoxide, releasing cytotoxic iron. The toxic effects of hemoglobin/hemin include release of redox-active iron, depletion of glutathione, and production of free radicals [14]. To avoid the toxicity of hemoglobin/hemin, these substances are cleared from the extracellular space via binding to haptoglobin and hemopexin or via phagocytosis by microglia/macrophages.

Haptoglobin, an abundant protein in blood plasma, has the ability to bind hemoglobin. In the brain, it is produced and released by oligodendrocytes, thereby protecting the brain against extravascular hemoglobin toxicity [112]. Expression of haptoglobin is increased around the hematoma in animal models of ICH. Haptoglobin-deficient mice are more vulnerable to ICH-induced brain injury, and mice with haptoglobin overexpression are less susceptible to injury [112]. Sulforaphane, an NF-E2-related factor2 (Nrf2) activator, increases haptoglobin in the brain and reduces brain injury following ICH [112]. In addition, sulforaphane treatment increases expression of Nrf2-mediated antioxidant genes, such as catalase, superoxide dismutase, and glutathione*S*-transferase, in the brain after ICH [113]. The antioxidative effects of sulforphane are correlated with reduction of brain damage, measured by brain edema, blood–brain barrier impairment, cortical apoptosis, and motor deficits [114].

Peroxisome proliferator-activated receptorγ (PPARγ) plays an important role in augmenting phagocytosis and promoting hematoma absorption [36,37]. PPARγ is a ligand-dependent transcription factor that regulates the expression of several target genes, such as scavenger receptor CD36 [36,37]. CD36, a class B scavenger receptor, is important for phagocytic activity [115,116]. Treatment with PPARγ agonists, such as rosiglitazone and 15d-PGJ2, increases expression of CD36 and promotes phagocytosis of RBCs by microglia/phagocytes in both *in vitro* and *in vivo* models of ICH [36,37], and anti-CD36 antibodies prevent PPARγ agonist-induced increases in phagocytosis [37]. In addition, in an animal model, treatment of ICH with PPARγ agonists accelerated hematoma resolution and reduced neurological deficits [36,37].

ROS are produced after ICH and contribute to ICH pathogenesis [4,5,13,34]. In addition, phagocytosis generates a large amount of ROS that can damage macrophages and neurons. PPARγ also plays an important role in protecting microglia/macrophages from oxidative damage via upregulation of the antioxidant catalase [36,37]. PPARγ agonists upregulate catalase expression in microglia *in vitro* and *in vivo* after ICH, enhance phagocytosis *in vitro* and increase hematoma absorption *in vivo*[37]. The PPARγ-mediated upregulation of catalase reduces oxidative stress, as demonstrated by a significant reduction of extracellular hydrogen peroxide in cultured microglia [37]. Phagocytosis of RBCs by microglia is also enhanced by upregulation of catalane, as demonstrated by the finding that addition of exogenous catalane to the culture media promotes phagocytosis [37].

PPARγ can also induce neuroprotection after ICH via anti-inflammatory effects. In both *in vitro* and *in vivo* experiments, PPARγ activators reduced expression of proinflammatory genes, including TNF-α, IL-1β,MMP9, and inducible nitric oxide synthase [37]. PPARγ is known to inhibit DNA binding of NF-κB [34,55], which controls expression of proinflammatory cytokines and enzymes, suggesting that the anti-inflammatory effect of PPARγ probably results from inhibition of NF-κB [37,117].

### TLR4 signaling in hematoma resolution

**Figure 1:**
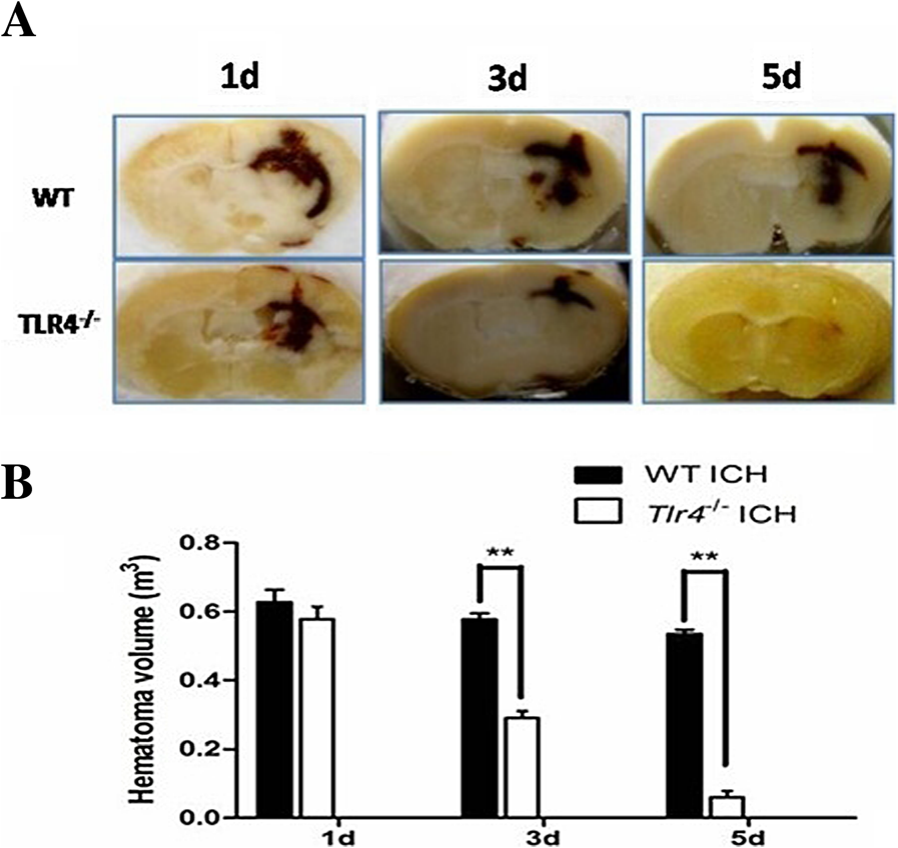
**TLR4 knockout results in faster hematoma resolution.** (**A**) Representative brain sections from WT and TLR4-knockout mice at 1, 3, and 5 days after intrastriatal injection of blood. (**B**) Compared with WT mice, TLR4-knockout mice had significantly smaller hemotomas at 3 and 5 days after ICH. Hematoma volume was measured on coronal slices (2 mm thick) using image analysis software. Scale bar: 1 mm. **P < 0.05 versus WT mice.

Microglia/macrophages express the scavenger receptor CD36, which has been reported to assist in phagocytosis-mediated removal of RBCs after ICH [37]. PPARγ agonists can promote phagocytosis of RBCs by microglia/phagocytes through upregulation of CD36 [36,37]. In addition, PPARγ agonists suppress the subarachnoid hemorrhage-induced inflammatory response by inhibiting TLR4 signaling [118]. Knockout of TLR4 results in upregulation of CD36 in the perihematomal region [31], suggesting that the TLR4 signaling pathway could play a role in hematoma resolution. In agreement with this hypothesis, we found that hematoma resolved significantly faster in TLR4-knockout mice than in WT mice, as shown in Figure 1. Specifically, hematoma started to resolve at 3 days after ICH and almost completely resolved by 5 days. These data demonstrate that the TLR4 signaling pathway is involved in hematoma resolution after ICH, and the underlying mechanisms are currently under investigation.

## Conclusions and perspectives

Increasing evidence has shown that TLR4 signaling plays important roles in ICH-induced inflammatory brain injury by stimulating activation of microglial cells, infiltration of leukocytes, and production of cytokines and chemokines [28,31]. The TLR4 signaling pathway involved in ICH-induced inflammatory injury includes ligands (e.g., heme), TLR4 itself, and its downstream pathways, including adaptor proteins (MyD88 and TRIF) and transcription factors, such as NF-κB [28]. Therefore, TLR4 and its signaling pathways are potential targets for developing effective medical treatment of ICH.

There are many challenges to be overcome before inhibition of TLR4 signaling can be used in the prevention and treatment of ICH. Though inhibition of TLR4 signaling by anti-TLR4 antibodies or deletion of TLR4 genes can effectively reduce ICH-induced neurological deficits in mice [28], specific TLR4 antagonists that inhibit TLR4 signaling have not been investigated in models of ICH. In addition, a diverse range of endogenous ligands may activate TLR4 signaling to trigger the inflammatory response that is critical in ICH-induced brain injury. However, it remains unclear how these ligands activate TLR4 signaling, which ligands are the most critical, how TLR4 receptors recruit adaptor proteins and activate transcription factors, and whether TLR4 signaling pathways are similar across cell types (e.g., microglia versus leukocytes). Whether TLR4 interacts with other TLRs in ICH-induced inflammatory injury requires further investigation. Better understanding of the roles of TLR4 signaling in ICH will facilitate development of ICH treatments.

Brain injury following ICH is triggered by the presence of intraparenchymal blood. The mechanisms of ICH-induced brain injury are numerous, including cytotoxic, oxidative, and inflammatory pathways. It would be beneficial to develop a medical treatment that promotes hematoma resolution and inhibits cytotoxic, oxidative, and inflammatory insults following ICH. For example, PPARγ activators reduce ICH-induced brain injury by improving hematoma resolution and reducing oxidative injury [36,37]. Another promising intervention is inhibition of TLR4 signaling, which also promotes hematoma resolution (see Figure 1) and inhibits ICH-induced inflammation [28]. However, the molecular mechanisms underlying hematoma resolution and cytotoxic, oxidative, and inflammatory injury following ICH remain elusive. Further research into the complex mechanisms involved in ICH pathogenesis will facilitate identification of novel therapeutic targets.

## Abbreviations

Hb: Hemoglobin; HMGB1: High-mobility group box 1 protein; HO: Heme oxygenase; ICH: Intracerebral hemorrhage; IFN-β: Interferon β; IL-1β: Interleukin-1β; LPS: Lipopolysaccharide; MyD88: Myeloid differentiation factor 88; NF-κB: Nuclear factorκB; Nrf2: NF-E2-related factor2; PPARγ: Peroxisome proliferator-activated receptorγ; RBC: Red blood cell; TIR: Intracellular Tollinterleukin 1 receptor; TIRAP: TIR domain-containing adaptor protein; TLR: Toll-like receptor; TNF-α: Tumor necrosis factorα; TRAM: TRIF-related adaptor molecule; TRIF: TIR domain-containing adaptor-inducing interferon; WT: Wild type.

## Competing interests

The authors declare that they have no competing interests.

## Authors’ contributions

All authors read and approved the final manuscript. FH collected literatures and reviewed the literatures. WPF reviewed the literatures. WYC proofreaded and corrected the manuscript. ZY revised the manuscript. YQW wrote the manuscript and approved the final version of the manuscript.
